# Older adults’ mental health during humanitarian crisis

**DOI:** 10.1192/j.eurpsy.2023.1140

**Published:** 2023-07-19

**Authors:** C. A. De Mendonca Lima

**Affiliations:** EPA Section of Old Age Psychiatry, Mézières, Switzerland

## Abstract

**Introduction:**

A humanitarian crisis is defined as a singular event or a series of events that are threatening in terms of health, safety or wellbeing of a community or group of individuals, and require action that is usually urgent and often non-routine. Examples of such crisis are wars, natural disasters, epidemics and forced immigration. There is an urgent need of an international commitment to planning for humanitarian emergencies that include individual and community psychosocial support for older adults with mental health conditions. The current lack of inclusion for these older adults in humanitarian response is dramatic and constitute a clear violation of their Human Rights.

**Objectives:**

The World Psychiatric Association Section of Old Age Psychiatry and the International Psychogeriatric Association are working together since 2020 to promote the older adults’ Human Rights. Articles, position statements, seminars, sympsoia and congress were produced. The ultimate common goal is to support the adoption of an UN Convention on the Human Rights of the Older Persons that include the promotion and protection of the mental health of these persons.

**Methods:**

Input to the Independ Expert on Older Adults at the OHCHR who prepared official repports presented during the UN General Assembly in 2022.

Publication of articles, organization of seminars, symposia and congress

**Results:**

The main documents pulblished will be presented as well the template of the next Position statement on Older Adult’s mental health during Humanitarian Crisis

**Conclusions:**

Humanitarian actors must provide assistance in accordance with the principles of humanity, neutrality and impartiality. Promoting and ensuring compliance with these principles are essential elements of effective humanitarian coordination, in respect of the Human Rights principles, in particular when vulnerable people such older adults with mental health conditions are involved.

**Disclosure of Interest:**

None Declared

